# Microcystin Prevalence throughout Lentic Waterbodies in Coastal Southern California

**DOI:** 10.3390/toxins9070231

**Published:** 2017-07-22

**Authors:** Meredith D. A. Howard, Carey Nagoda, Raphael M. Kudela, Kendra Hayashi, Avery Tatters, David A. Caron, Lilian Busse, Jeff Brown, Martha Sutula, Eric D. Stein

**Affiliations:** 1 Southern California Coastal Water Research Project, 3535 Harbor Boulevard, Suite 110, Costa Mesa, CA 92626, USA; jeffb@sccwrp.org (J.B.); marthas@sccwrp.org (M.S.); erics@sccwrp.org (E.D.S.); 2 San Diego Regional Water Quality Control Board, 2375 Northside Drive, Suite 100, San Diego, CA 92108, USA; carey.nagoda@waterboards.ca.gov; 3 Department of Ocean Sciences, University of California, Santa Cruz, 1156 High Street, Santa Cruz, CA 95064 USA; kudela@ucsc.edu (R.M.K.); khayashi@ucsc.edu (K.H.); 4 Department of Biological Sciences, University of Southern California, 3616 Trousdale Parkway, Los Angeles, CA 90089-0371, USA; tatters@usc.edu (A.T.); dcaron@usc.edu (D.A.C.); 5 German Federal Environmental Agency, Umweltbundesamt, Wörlitzer Platz 1, 06844 Dessau, Germany; lilian.busse@uba.de

**Keywords:** cyanotoxins, cyanobacteria, microcystins, cylindrospermopsin, anatoxin-a, California, lakes, estuaries, SPATT

## Abstract

Toxin producing cyanobacterial blooms have increased globally in recent decades in both frequency and intensity. Despite the recognition of this growing risk, the extent and magnitude of cyanobacterial blooms and cyanotoxin prevalence is poorly characterized in the heavily populated region of southern California. Recent assessments of lentic waterbodies (depressional wetlands, lakes, reservoirs and coastal lagoons) determined the prevalence of microcystins and, in some cases, additional cyanotoxins. Microcystins were present in all waterbody types surveyed although toxin concentrations were generally low across most habitats, as only a small number of sites exceeded California’s recreational health thresholds for acute toxicity. Results from passive samplers (Solid Phase Adsorption Toxin Tracking (SPATT)) indicated microcystins were prevalent throughout lentic waterbodies and that traditional discrete samples underestimated the presence of microcystins. Multiple cyanotoxins were detected simultaneously in some systems, indicating multiple stressors, the risk of which is uncertain since health thresholds are based on exposures to single toxins. Anatoxin-a was detected for the first time from lakes in southern California. The persistence of detectable microcystins across years and seasons indicates a low-level, chronic risk through both direct and indirect exposure. The influence of toxic cyanobacterial blooms is a more complex stressor than presently recognized and should be included in water quality monitoring programs.

## 1. Introduction

The global proliferation of toxin producing cyanobacterial blooms has gained international attention in recent years [1,2,3,4,5,6]. These increases have been attributed to a wide variety of environmental factors including nutrient pollution, increased temperature, salinity, water residence time, vertical stratification, and pH, many of which will likely be exacerbated by climate change [6,7,8,9,10,11,12,13,14].

Cyanotoxins pose a significant risk for humans, livestock, pets, and wildlife, causing illness and mortality [15,16,17,18,19,20,21,22,23,24,25]. Cyanotoxin poisoning in pets and livestock is significantly under-recognized and under-reported [16,23,25]. Due to the adverse health effects associated with cyanotoxins, and the growing recognition that cyanobacterial blooms can severely impact water quality [26,27], health advisory thresholds for some cyanotoxins have been developed by many states, including California. The U.S. Environmental Protection Agency has recently released health advisory thresholds for cyanotoxins in drinking water [28,29] and proposed draft thresholds for recreational water and swimming activities [30]. The USGS has recently prioritized 12 cyanotoxins as Tier 1, the highest priority for inclusion in ambient water monitoring in the U.S. (an additional three were listed at intermediate or low priority) [31]. 

Despite recognition of this growing threat, the extent and magnitude of cyanobacterial blooms in California are poorly characterized, particularly in heavily populated regions such as coastal southern California. Across the continuum of aquatic habitats, lentic waterbodies, such as lakes, reservoirs, depressional wetlands and coastal lagoons, provide conditions that readily support the proliferation of cyanobacterial blooms as they typically include ample supply of nutrients, calm water and stratified conditions, high irradiance and warm water temperatures. In southern California, these lentic habitats are often associated with or downstream of urban and agricultural areas, and therefore subject to further risk of cyanobacteria proliferation due to increased anthropogenic nutrient inputs via point- and non-point source runoff.

Hydrologic alteration is pervasive throughout southern California watersheds, with increased peak flows and altered base flow correlated with total impervious cover [32,33]. Eutrophic habitats are ubiquitous throughout the region. Fetscher et al. [34] found that 60% of southern California streams had elevated stream algal biomass, while McLaughlin et al. [35] found that 83% of southern California coastal lagoons had excessive macroalgal growth. Comprehensive lake surveys have not been undertaken, but regulatory actions to reduce nutrient loads have been enacted for over 13 lakes, and are pending in another 12 lakes that are listed as impaired for nutrients and eutrophication [36].

There are few published studies on cyanotoxins in southern California lentic waterbodies. However, microcystin production was documented from benthic cyanobacteria in several reservoirs [37], and in wadeable streams throughout the State [38]. California Statewide assessments revealed that benthic algae in wadeable streams were a source of cyanotoxin production and supported a high occurrence of potentially toxigenic taxa [38]. Over 90% of stream kilometers in California supported potential toxin-producing genera and 23% supported toxin-producing species [38]. Cyanotoxin analysis from that assessment detected microcystins in 33% of sites statewide, lyngbyatoxin in 21% of sites, and saxitoxin or anatoxin-a was detected in 7% of sites [38]. Cylindrospermopsin and nodularin were not detected in the survey [38]. Similarly, cyanobacteria were ubiquitous in a screening assessment of 30 lakes, depressional wetlands and coastal lagoons in southern California, with *Microcystis* spp. dominant in 96% of study sites [39]. Quantification of cyanotoxins in that study were limited, however, microcystins were detected in high concentrations at 10% of sites that exceeded both the California recreational health thresholds, and the World Health Organization guidance for human health. Cylindrospermopsin and anatoxin-a were not detected at any sites. The community composition was dominated by potentially toxic cyanobacteria (particularly *Microcystis* spp.); therefore, these findings generated interest in further characterizing cyanotoxins, and specifically microcystin prevalence, across lentic waterbodies in southern California. 

Concerns of ecological and human health risks of cyanotoxins also extend beyond freshwater habitats and into the land-sea interface. Multiple studies in central California and one in Washington State have documented microcystins in marine outflows, with direct impacts in marine ecosystems far downstream of their biological origin [40,41,42,43,44,45,46,47]. The mass mortality of more than 30 marine sea otters in Monterey Bay was attributed to microcystin intoxication from ingestion of contaminated shellfish [45]. Microcystin production originated in Pinto Lake, a eutrophic water body that experiences frequent cyanobacterial blooms and drains to Monterey Bay via a 15-km segment of the Pájaro River [45,48]. Extensive watershed studies conducted in Monterey Bay, California and Washington State showed that this downstream transport of microcystins was a persistent and prevalent issue throughout the watershed (not just from a single waterbody) and that the toxins accumulated in marine shellfish [42,43,47]. These studies underscore an important role of rivers and streams as conduits that can not only produce cyanotoxins, but can also transport intact toxins from inland waters to downstream marine and estuarine waterbodies. 

Given the severe and ubiquitous nature of harmful cyanobacterial blooms and cyanotoxins demonstrated by these studies, surveillance and monitoring have become critical for the protection of public health. Traditional monitoring programs for these toxins typically rely on discrete sampling (“grab” samples) from a particular site or sites. These methods are inherently biased if the sampling does not capture the spatial and temporal variability of the system, and ephemeral or episodic events. In response to this challenge, a passive sampling method, Solid Phase Adsorption Toxin Tracking (SPATT), has been developed to monitor toxin concentrations of cyanobacterial and algal toxins in marine, brackish and freshwater environments [45,48,49,50]. These toxins have often been detected using SPATT when simultaneous discrete water samples have failed to detect them in a given waterway or waterbody, making SPATT a more sensitive indicator of the prevalence of toxins than traditional discrete samples [42,48,49]. SPATT was therefore used in the current study as an additional assessment tool to provide insight into the overall toxin prevalence and to provide a time-integrative toxin sample. 

The objectives of the current study were to: (1) determine the prevalence of microcystins in lentic waterbodies in southern California to evaluate if they should be included in routine water quality monitoring programs; and (2) evaluate the applicability of a passive sampling technology (SPATT) to detect toxins in monitoring and assessment programs. Microcystins were the focus of the current study due to the dominance of *Microcystis* spp. in previous screening assessments [38,39], and because microcystins are the most common cyanotoxin detected in lakes in the U.S. [51]. Three separate field surveys were conducted: (1) an ambient probability based assessment of depressional wetlands (2011–2013); (2) a screening assessment of lakes, reservoirs and coastal lagoons determined to be at significant risk for blooms in 2013; and (3) an ad hoc bloom event response survey in 2014. 

## 2. Results

### 2.1. Depressional Wetlands Assessment

**Figure 1:**
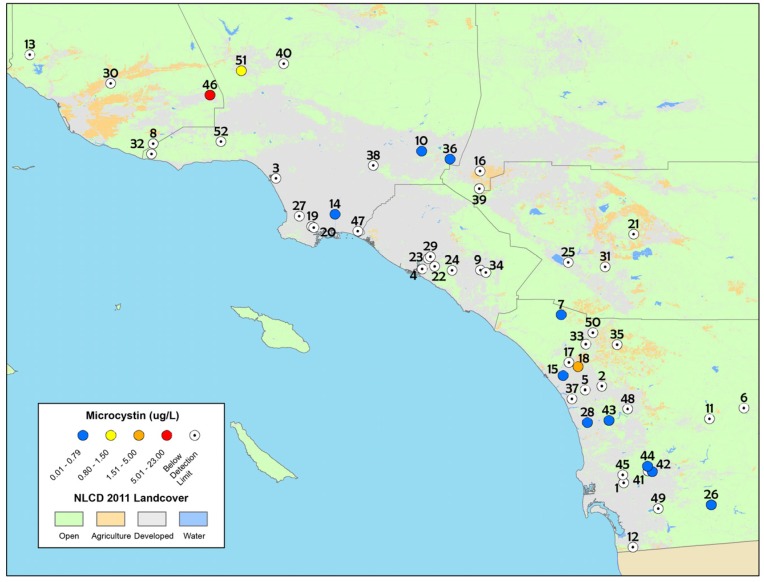
Map of particulate microcystin concentrations detected from discrete samples in the spring depressional wetlands assessment, 2011–2013. The numbers on the map correspond to sites in Table 1. The white circles with a black dot in the center indicate sites that were below the method limit of detection while blue circles indicate sites that had microcystin concentrations ranging from 0.01 to 0.79 µg L^−1^. The yellow, orange and red circles indicate microcystin concentrations that exceeded the California recreational health thresholds for microcystins (0.8 µg L^−1^).

Particulate microcystins were detected at 25% of sites examined during the field study (Table 1) over the whole sampling period (2011–2013), though the percentage of sites with microcystins detected varied widely from year to year, as did concentrations of microcystins (Table 2, Figure 1). Particulate microcystins were detected from 12.5% of all sites sampled in 2011 and 2013 and 47% of all sites sampled in 2012. The range of particulate microcystin concentrations varied from below detection to 2.5 µg L^−1^ in 2011, up to 0.45 µg L^−1^ in 2012, and up to 22 µg L^−1^ in 2013. Interestingly, the highest percentage of positive sites was observed in 2012, although concentrations of particulate microcystins did not exceed the California recreational health thresholds (0.8 µg L^−1^ OEHHA, 2012; 1.0 µg L^−1^ http://www.mywaterquality.ca.gov/monitoring_council/cyanohab_network/docs/triggers.pdf). In contrast, there were only two sites with microcystins detected during 2013, but, concentrations at both sites exceeded the California recreational health thresholds. Saxitoxins were detected at very low concentrations (<0.4 µg L^−1^) at only one site during the 2012–2013 assessment (Santee Lakes Recreation Preserve Lake, site number 44 on Table 1 and Figure 1).

Statistical analysis revealed that chlorophyll-a was not a significant predictor of microcystin concentration (*p*-value, 0.9), neither across years nor for any individual year. Additionally, no statistically significant relationships were identified between microcystin concentrations and environmental variables, including alkalinity (*p*-value = 0.7), total nitrogen (*p*-value = 0.2), total phosphorus (*p*-value = 0.7), pH (*p*-value = 0.7), elevation (*p*-value = 0.4), conductivity (*p*-value = 0.8), and temperature (*p*-value = 0.7). Similarly, the landscape disturbance indicators (percentage of agriculture, urban, impervious cover and road density) also were not identified as statistically significant predictors of microcystins, regardless of the area surrounding the wetland that was used in the analysis (30 m, 150 m, 500 m, 1000 m and 3000 m). The three condition indices, macroinvertebrates index, benthic diatom index and CRAM, were also not statistically significant predictors of microcystins (*p*-values of 0.07, 0.3, and, 0.1 respectively).

**Figure 2:**
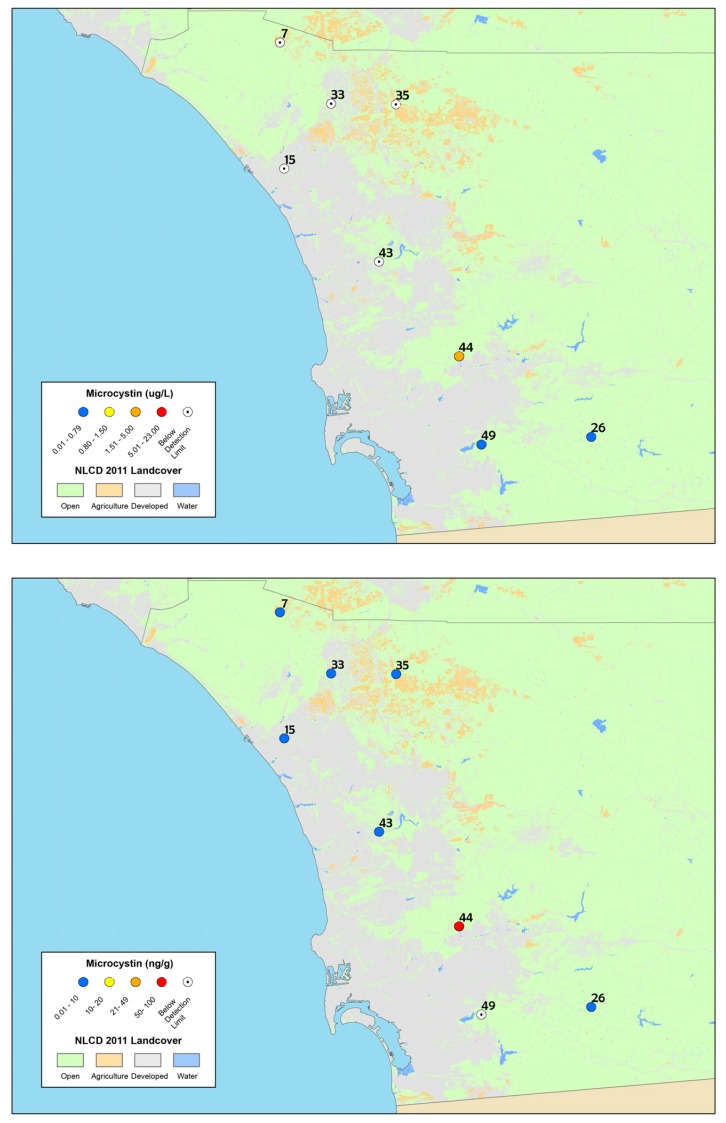
Maps of: particulate microcystin results (**top**); and time-integrated dissolved microcystin results from SPATT samples (**bottom**), collected in summer and fall 2012 from the depressional wetlands assessment sites in San Diego County. The site numbers correspond to Table 1 and the legend is the same as Figure 1. The highest concentrations are shown for discrete samples (sites were sampled twice).

The depressional wetlands sites sampled in the spring of 2012 in San Diego County were re-sampled during the summer and early fall (Figure 2) and time-integrating passive samplers (SPATT) were also deployed. During the spring 2012 assessment, particulate microcystins were detected at 60% of the sites in San Diego County based on discrete samples only (Table 3). During the summer and fall seasons when these sites were re-sampled, microcystins were detected at only 29% of sites based on discrete sample results, while time-integrated SPATT results detected dissolved microcystins at 83% of sites (Figure 2, Table 3). The results of SPATT and discrete samples from all seasons in 2012 are summarized in Table 3 and shown in Figure 2.

Particulate saxitoxins were detected at one site (equivalent to 10% of survey sites) during both the spring and summer (Santee Lakes Recreation Preserve lake, site number 44 on the map in Figure 1 and Figure 2), and the concentrations were extremely low (<0.04 µg L^−1^).

The most common microcystin congener detected from the SPATT samples was MCY-LR, followed by MCY-RR and MCY-LA. MCY-YR was not detected in the samples (Supplementary Materials, Table S1).

### 2.2. Surveys of Lakes, Reservoirs, and Coastal Lagoons

#### 2.2.1. Screening Assessment Survey 2013

The screening assessment survey of lakes, reservoirs, and coastal lagoons yielded results that, similar to the depressional wetlands assessment, revealed widespread occurrence of micorocystins. Results from time-integrated SPATT samplers indicated that all sites had measurable dissolved microcystins during at least one of the three sampling visits throughout the field survey and seven sites had measurable dissolved microcystins throughout the field survey (Table 4, sites in bold font). In contrast, particulate microcystin results obtained from discrete samples indicated that 26% of the sites had measurable particulate microcystins during the field survey. Two of these sites exceeded the recreational action thresholds for California (OEHHA, 2012; http://www.mywaterquality.ca.gov/monitoring_council/cyanohab_network/docs/triggers.pdf). Morena Reservoir had 23.6 µg L^−1^ of microcystins detected, indicating the Tier II, Danger threshold and Vail Lake had 2.1 µg L^−1^, indicating the Caution trigger threshold. 

**Figure 3:**
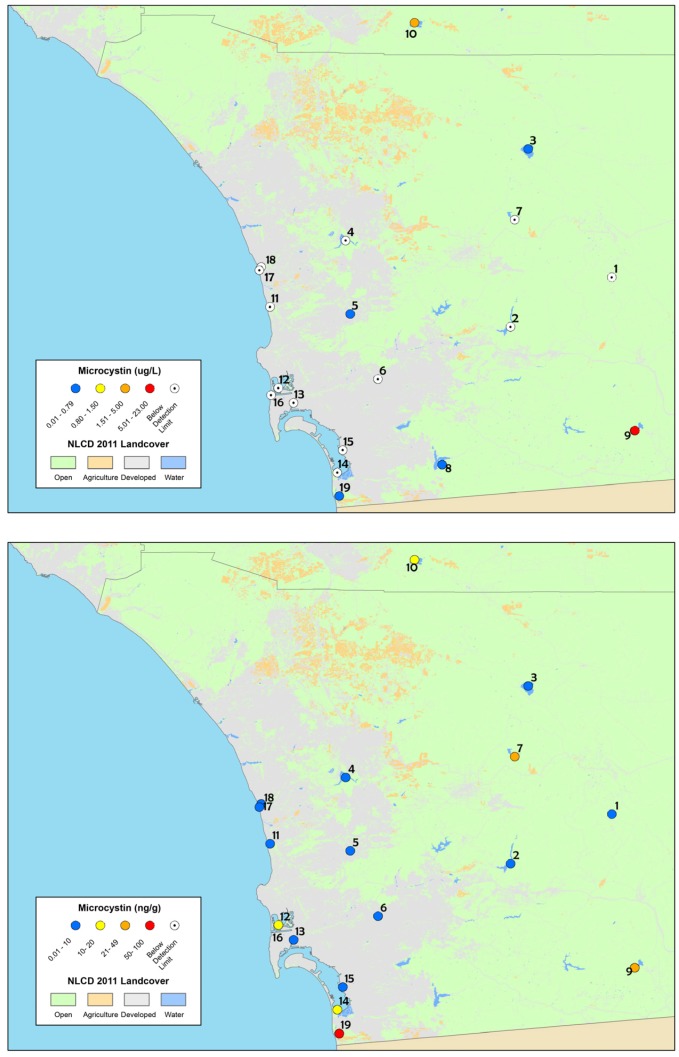
Maps of: discrete sample results (**top**); and SPATT sample results (**bottom**), for microcystins collected from the screening assessment survey of lakes, reservoirs and coastal lagoons in 2013. The site numbers correspond to Table 2 and the legend is the same as Figure 1.

Particulate microcystin concentrations ranged from below the detection limit of the method (bd) to 23.6 µg L^−1^ (Table 4; Figure 3) and below the detection limit (bd) to 100.8 ng g^−1^ for dissolved microsystins detected from SPATT samples (Table 4; Figure 3). 

Statistical analysis revealed that chlorophyll-a was a statistically significant predictor of microcystin concentration (*p*-value = 0.004) during the screening assessment survey of lakes, reservoirs and coastal lagoons. There were no statistically significant environmental predictors (such as alkalinity or nutrients) of microcystins (*p*-values > 0.05). The pigment results were similar in that neither phycocyanin nor phycoerythrin was a statistically significant predictor of microcystins (*p*-values > 0.05). 

MCY-RR was the most commonly detected dissolved microcystin congener of the four analyzed, was detected at every site, and was present in 64% of the SPATT sample results (Supplementary Materials, Table S2). MCY-LR was only detected at six sites and MCY-LA at five sites (out of the total of 18 for which there are SPATT results). MCY-YR was not detected at any of the sites from the SPATT samples. Particulate microcystin results were positive at only four sites (out of 19 surveyed) for any of the nine microcystin congeners analyzed (Supplementary Materials, Table S3). Within those four sites, MCY-LR and MCY-YR were the most common congeners detected at three of the sites. Two sites exceeded the California recreational action thresholds for microcystins, Vail Lake (2.1 µg L^−1^) and Morena Reservoir (two time points, August and September, 6.1 µg L^−1^ and 23.6 µg L^−1^, respectively). 

#### 2.2.2. Ad Hoc Bloom Event Response Survey

**Figure 4:**
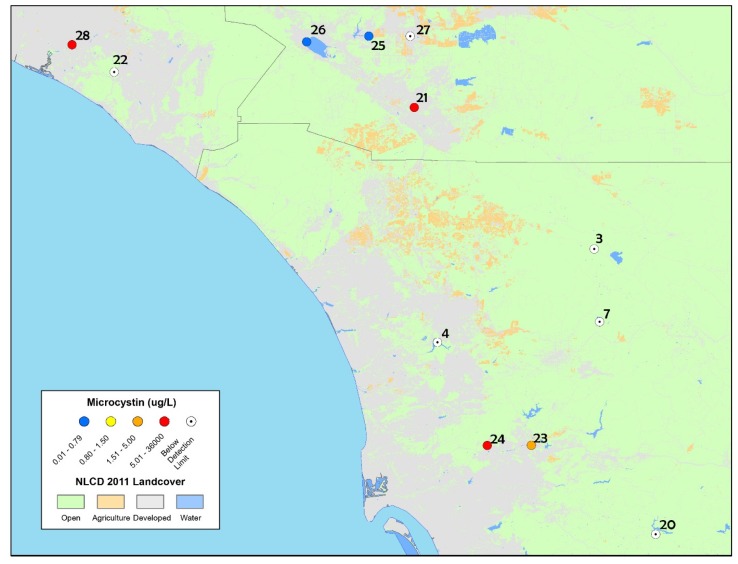
Map of discrete sample results collected from the ad hoc bloom event response survey in 2014 in San Diego, Riverside and Orange Counties (including one sample collected in 2015 from San Joaquin Marsh). The highest concentrations of microcystins are reported for sites that were re-sampled (and therefore have multiple toxin results).

Microcystins were detected in half of the sites analyzed for toxins during the ad hoc bloom event response survey in 2014, with concentrations ranging from below detection to 36,549 µg L^−1^ (results of the discrete samples are shown in Figure 4 and Table 5). Nodularin was not detected in any of the discrete samples. Four sites exhibited microcystin concentrations that exceeded the California recreational health thresholds (OEHHA, 2012; http://www.mywaterquality.ca.gov/monitoring_council/cyanohab_network/docs/triggers.pdf). The results from the San Joaquin Marsh sample were the highest detected within southern California to date (total microcstyins = 36,549 µg L^−1^) from a whole water sample (i.e., excluding scum and foam samples). Particulate microcystins were also detected at three other sites: Harveston Lake, Lindo Lake and Santee Lake (concentrations of 10 µg L^−1^, 2.5 µg L^−1^, and 11.7 µg L^−1^, respectively). All are highly used recreational sites. These concentrations are likely underestimates of the total microcystins present since only the particulate fraction was measured.

Additional cyanotoxins, cylindrospermopsin and anatoxin-a, were analyzed from samples collected at three lakes, Canyon Lake, Lake Elsinore and Lake Menifee (site numbers 25, 26 and 27, respectively, in Figure 4). Cylindrospermopsin was detected at all three lakes, at concentrations that exceeded the California recreational health thresholds (OEHHA, 2012; http://www.mywaterquality.ca.gov/monitoring_council/cyanohab_network/docs/triggers.pdf). Total cylindrospermosin concentrations detected were 2.7 µg L^−1^ at Canyon Lake, 4.1 µg L^−1^ at Lake Elsinore, and 2.9 µg L^−1^ at Lake Menifee (CA trigger levels for cylindrospermopsin are 1 µg L^−1^ for caution, 4 µg L^−1^ for warning and 17 µg L^−1^ for danger). Total anatoxin-a was detected at Canyon Lake (4.6 µg L^−1^), and Lake Menifee (3.6 µg L^−1^), while Lake Elsinore was below the limit of detection. All detectable concentrations exceeded the California recreational health thredholds (OEHHA, 2012; http://www.mywaterquality.ca.gov/monitoring_council/cyanohab_network/docs/triggers.pdf). The detailed results of individual microcystin congeners are summarized in Supplementary Materials, Table S4. MCY-LR and MCY-RR were the most commonly detected congeners. 

The potentially toxic cyanobacterial taxa were identified to genus level and where possible to species level and the results are summarized in Table 5. *Microcystis* sp., *Cylindrospermopsis* sp., and *Anabaena* sp. were the three most common genera identified in 40–50% of the sites. The species that were identified included *Cylindrospermopsis raciborskii*, *Anabaena variabilis*, *A. spiroides*. These results indicated the potential for many types of cyanotoxins to be present including microcystins, saxitoxin, cylindrospermopsin, and anatoxin-a.

## 3. Discussion

### 3.1. Microcystin Prevalence 

Microcystins were detectable and prevalent in all lentic waterbody types surveyed across all land use types in southern California. Particulate microcystin concentrations were generally low across most habitats, exceeding California recreational health thresholds for acute toxicity at only a small number of sites during the depressional wetlands assessment and the screening assessment survey of 2013, similar to findings in the California Sacramento-San Joaquin Delta and San Francisco Bay estuary [44,52,53]. However, the number of waterbodies where microcystins were present was underestimated by discrete particulate samples based on the high number of samples and sites where dissolved microcystins were detected by SPATT samples. The persistence of detectable microcystins across multiple years and seasons indicates a low-level, chronic presence of microcystins in these waterbodies, similar to findings in Monterey Bay watersheds [42]. Chronic exposure of microcystins can have human and wildlife health implications [15,23,54,55,56,57,58,59] and can be transported into riparian food webs [60] and marine shellfish [42,43,45,46,47,61,62]. The sites sampled in this study were chosen due to the designed beneficial uses that include human and wildlife exposure (such as drinking water supply, agricultural water supply and both contact and non-contact recreation) [63]. In contrast to the assessment surveys, the ad hoc bloom event response survey indicated microcystins at half of the sites analyzed, and a quarter of sites had very high acute concentrations, 3–45,000-fold higher than the recreational health thresholds (0.8 µg L^−1^; OEHHA, 2012; 1.0 µg L^−1^ http://www. mywaterquality.ca.gov/monitoring_council/cyanohab_network/docs/triggers.pdf), indicating a high risk for immediate impacts to human, wildlife and domestic pet health. Currently, cyanotoxins are not included in routine water quality monitoring programs in California, but cyanotoxin analysis is conducted when visible blooms are reported to the State. The results of the current study detected microcystins (both dissolved and particulate) in waterbodies that had no visible cyanobacterial blooms indicating the current bloom event response efforts in California may be insufficient to protect public health. Cyanotoxins should be added to routine water quality monitoring programs in southern California to ensure protection of public health.

Microcystins produced in freshwater systems have been shown to cause effects far downstream of their biological origin, have been detected in downstream coastal receiving waters and near-shore marine waters and have accumulated in marine shellfish [42,43,45,46,47,61,62]. Multi-year studies of the Monterey Bay watershed have shown that downstream transport of microcystins is a persistent and prevalent issue throughout the watershed [42]. Similar downstream transport of microcystins from a freshwater lake into marine shellfish has been documented in Washington State [47]. These studies underscore an important role of rivers as conduits that can transport intact toxins from inland waters (e.g., lakes, reservoirs and wetlands) to downstream marine and estuarine waterbodies. The widespread and chronic production of microcystins across all waterbody types and the detection of microcystins in all coastal lagoon systems sampled determined from the current study, as well as other studies documenting the transport of toxins from inland to marine waterbodies [38,42,43,45,46,47], suggest microcystins should be added to the list of biotoxins routinely monitored in California coastal waters and marine shellfish.

Summer and fall seasons have been identified as the most conducive to cyanobacteria growth, bloom formation and toxin production [42,53,64]. Interestingly, the San Diego County depressional wetlands sites yielded a higher percentage of microcystins detected in spring (Table 3; 60%), compared with the summer and fall seasons (Table 3; 29%). Similar seasonal differences were observed in the Monterey Bay watershed, with spring providing the highest concentrations of microcystins in some rivers [42]. Our findings indicate that the occurrence of microcystins, and the seasonality of toxin production in California should be examined more comprehensively in future studies. We observed differences in the prevalent microcystin congeners within different waterbody types. For example, MCY-YR was not detected in the depresssional wetlands assessment or the 2013 screening assessment survey, but was detected in the ad hoc survey. These differences are likely due to differences in cyanobacterial species composition, the details of which are beyond the scope of this study. Tatters et al. [62] identified the prevalent potential toxin-producing cyanobacteria genera and species in estuaries and coastal lagoons along the coast of the Southern California Bight. Characterization of the prevalent southern California cyanobacteria species and corresponding toxin profiles should be the focus of future studies.

#### Chlorophyll-a as a Screening Indicator for Microcystin

Chlorophyll-a has been shown to be a meaningful screening variable in lakes at risk for cyanotoxins, and it is less expensive, easier to collect and analyze (or remotely sense) than cyanotoxins directly. Chlorophyll-a has been linked to pollution management actions such as nutrient load reductions [3,48,65,66,67]. In this study, there was a significant correlation between chlorophyll-a and microcystin concentrations in lakes, reservoirs, and coastal lagoons. Therefore, it is feasible based on these study results that future monitoring could utilize chlorophyll-a as a way to screen for microcystins in these waterbody types. Future studies should determine if this relationship holds for other types of cyanotoxins. However, no such correlative relationship was identified in the depressional wetlands waterbody type. The lack of correlation could be attributed two factors. First, it is possible that a stronger relationship could be found between toxin concentrations and benthic chlorophyll-a, which was not assessed. Many of these depressional wetlands are shallow (<1 m) dominated by benthic algal and cyanobacteria mats rather than planktonic species [68]. Second, the study design, based on one-time discrete sample collection (commonly used to evaluate chemical contaminants), is not ideal for cyanobacterial toxins as they can be sporadically and ephemerally produced, and thus toxin production can change within short time periods. As discussed below, the discrete sample results were not a good indicator of toxin presence in these systems (see Section 3.2 SPATT as an assessment and monitoring tool). 

### 3.2. SPATT as an Assessment and Monitoring Tool

Traditional monitoring programs for cyanotoxins typically rely on discrete samples for toxin analysis, and health advisory thresholds have been based around this method of sampling. These methods can effectively measure toxin presence on the day and at the time of sample collection, but they are less effective at capturing ephemeral or episodic toxic events. In response to this challenge, a passive sampling method, SPATT, has been developed to monitor toxin concentrations in marine, brackish and freshwater environments. SPATT was first proposed for harmful algal bloom monitoring in 2004 as a means by which disadvantages associated with shellfish or other indicator organisms might be circumvented [50]. The approach has recently been refined as an assessment and monitoring tool to provide a time-integrated indicator of dissolved toxin presence with a waterbody [48,49,69,70,71]. SPATT has been shown to be a more sensitive indicator of toxin presence than discrete samples [42,48,49], however, it cannot currently be compared with health advisory thresholds established by California and US EPA. SPATT, was successfully used in this study as a monitoring and assessment tool to determine the prevalence and persistence of dissolved microcystins. The results indicated a high prevalence throughout the region, indicating the probability of a low-level but chronic exposure via direct as well as indirect pathways and indication that dissolved microcystins are prevalent and should be included in monitoring and event response programs. 

### 3.3. Other Cyanotoxins and Multiple Stressors

Cyanobacteria genera and species identifications indicated a high risk for multiple cyanotoxins to be routinely produced and co-occur in these systems. The co-occurrence of multiple cyanotoxins at a single location has been documented in other studies, both within and outside of the U.S. [62,72,73,74,75,76] and in lagoons and estuaries within our study region in southern California [62]. There were a wide variety of potential toxin producing cyanobacteria identified in our study, but the most common genera were *Microcystis* sp., *Cylindrospermopsis* spp., and *Anabaena* spp., suggesting microcystins cylindrospermopsin, anatoxins and saxitoxin could be widespread. As such, a subset of lake samples was analyzed for additional cyanotoxins, and the results revealed the first detection of anatoxin-a from lakes in southern California as well as simultaneous detection of multiple cyanotoxins. 

Lake Elsinore, Lake Menifee, and Canyon Lake were tested for additional cyanotoxins and were positive for 2–3 cyanotoxins. Lake Menifee and Canyon Lake had concentrations of cylindrospermopsin and anatoxin-a that exceeded recreational health thresholds. Cylindrospermopsin concentrations detected in Lake Elsinore exceeded recreational health thresholds. Previous studies of cyanobacteria species identification in 2003 and 2010 in Lake Elsinore indicate the potential for a historical presence of multiple cyanotoxins, since potential toxin producing species identified in 2003 and 2010 included *Cylindrospermopsis raciborskii*, *Cylindrospermopsis c.f. catemaco*, *Aphanizomenon*, *Pseudanabaena limnetica*, *Pseudanabaena c.f. acicularis*, *Pseudanabaena catenata*, and *Planktothrix agardhii* [77,78].

The combination of multiple cyanotoxin producing species identified, and the detection of multiple cyanotoxins simultaneously, highlights an important data gap in our understanding of the commonly occurring cyanotoxins, and cyanobacteria in these lentic systems. As with the interplay between acute and chronic risk, the interaction of multiple stressors on humans and other wildlife make the risk uncertain, because most recreational and drinking water health thresholds are typically based on exposures to single toxins and single organisms. The health consequences and implications of exposure to co-occurring cyanotoxins is poorly characterized. These biotoxins have different mechanisms of toxicity that could have synergistic effects and act as different, but additive, physiological stressors.

### 3.4. Conclusions and Recommendations

The dominance of cyanobacteria in the lentic waterbodies in the present study and the ubiquitous and persistent detection of microcystins in these heavily utilized aquatic habitats, suggest that cyanobacteria characterization and cyanotoxin detection should be included in routine and systematic monitoring programs of these systems. Future studies need to characterize the cyanobacteria species that are producing toxins and determine the concentration ranges routinely present in these systems. Passive sampling devices, such as SPATT, were successfully used in this study to determine prevalence of microcystins and should be included in future ambient monitoring and assessment programs. These sampling devices are particularly useful at capturing ephemeral events that traditional discrete samples do not capture, and therefore provide a more comprehensive view of cyanotoxins in a waterbody or region but are not at this time a replacement for traditional grab sampling, required for public health monitoring. 

The potential for bioaccumulation of multiple cyanotoxins into the food web suggests that the influence of toxic cyanobacterial blooms is a much more complex stressor than presently recognized and should be considered a high priority measurement to include in condition assessments, water quality monitoring programs and additional risk assessments. Both high acute concentrations and low chronic concentrations of cyanotoxins should be considered a predominant stressor within lentic waterbodies. Since many of these waterbodies and watersheds are ultimately connected to the coastal ocean, these studies underscore the importance of inland waters as potential conduits for transfer of freshwater toxins to the marine environment, as evidenced by the occurrence of these toxins within the marine food web [43,45,47].

## 4. Methods

### 4.1. Study Area

The field surveys were all conducted within the coastal regions of the Southern California Bight (SCB), which is an open embayment that stretches from Point Conception, California, to Cabo Colnette, Baja California. The climate of this region is Mediterranean, with an average annual rainfall range of 10 to 100 cm, concentrated largely over the winter months of December-March, followed by hot, dry summers [79]. The SCB is a highly developed urban environment with a heavily altered landscape due to the approximately 20 million residents and 100 million visitors to the area annually [80]. This urbanization has extensively altered the natural landscape, watersheds and waterbodies, and converted significant amounts of open land to impervious surfaces, thereby changing both the timing and the rate of runoff releases to downstream waterbodies [80,81,82,83]. The majority of the nutrient loading is derived from non-point sources, since municipal wastewater is discharged as point sources into ocean outfalls directly to the SCB [84]. The region has few natural lakes and open-water depressional wetlands. Their abundance on the landscape can be attributed to type-conversion of riverine and palustrine habitats to create drinking water and recreational reservoirs, and stormwater detention basins [85]. 

### 4.2. Depressional Wetlands Assessment

The first ever systematic condition assessment of southern California depressional wetlands was conducted as part of the regional monitoring and assessment program led by California’s Surface Water Ambient Monitoring Program (SWAMP) [68]. The goals of the condition assessment were to determine: (1) the extent and distribution; and (2) the condition of depressional wetlands; and (3) to identify major stressors (such as urban and agricultural contaminants, invasive species, nutrient loading etc.). Cyanotoxins were not the focus of the regional condition assessment but samples were collected opportunistically as part of the field sampling program associated with the assessment. Particulate material only (i.e., plankton in the water column) was collected for cyanotoxin analysis, along with chlorophyll-a samples, following the guidelines in the Standard Operating Procedures (SOP) [86]. This approach excluded analysis of dissolved toxins, and therefore, underestimated the total concentration of cyanotoxins in the water. 

#### 4.2.1. Site Selection and Sampling Approach for Discrete Samples

The sites were randomly selected using a probabilistic sampling design by the Generalized Random Tessellation Stratified (GRTS) technique [87] in a spatially balanced approach. The assessment was conducted from 2011–2013, and each site was revisited once (May/June) after the initial reconnaissance survey. The 52 sites are summarized and categorized in Table 1. 

Discrete samples were collected from composite surface water samples and a detailed version of the sample collection methodology is described in the SWAMP Depressional Wetlands Standard Operating Procedures [86]. Briefly, composite water samples were collected from 10 sampling “nodes” evenly spaced throughout the entire wetland perimeter. Field crews counted how many paces are required to walk the perimeter of the wetland at the water’s edge, then that number of paces was divided by 10, to yield the distance between adjacent sampling nodes. The field crews paced the perimeter of the wetland again, and placed an orange flag at each sampling node. They collected 200 mL sample from each sampling node, using an extended sampler device that allowed the collection of undisturbed samples approximately an arm’s length from the water’s edge, and placed the water into 2 L aluminum foil-covered bottles. Discrete samples were collected from the 2 L composite sample bottle after gentle mixing (inverted bottle upside-down five times) [86]. Particulate samples of chlorophyll-a and cyanotoxins were filtered onto 47 mm Whatman GF/F filters and frozen immediately in the field. Cyanotoxins were not the focus of the regional condition assessment, therefore the approach was to collect only filtered particulate fraction samples in the same way as chlorophyll-a samples [86] to ensure the samples would be collected with ease in a timely manner that allowed for completion of all of assessment samples. This collection approach also added a concentration step which allowed for a lower limit of detection. Additional water samples were collected and filtered through 0.45 µm polytetrafluoroethylene (PTFE) filters for later analysis of dissolved nutrients (ammonia, nitrate, nitrite, orthophosphate) and whole water samples were collected for total nitrogen, total Kjeldahl nitrogen (TKN), total phosphorus and alkalinity. All samples were frozen immediately in the field [86]. 

A more thorough cyanotoxin assessment was conducted in San Diego County in 2012 as both the particulate and dissolved fractions were measured. The 8 sites from the depressional wetlands assessment (sampled in May) were revisited twice between July and September (indicated by an asterisk (*) next to the site number in Table 1). Passive sampling devices, SPATT [48,49,50], were deployed continuously between site visits and were used as a screening assessment tool that provided time-integrated dissolved toxin samples of microcystin presence in the waterbodies. SPATT were deployed in 1 location of the wetland (the easiest to access and deploy) using a PVC tube and a rope tied to the SPATT sampler for approximately 1-month intervals between July and September 2012. Discrete samples were collected during each SPATT deployment and retrieval, for a total of two discrete sampling events per site. Discrete samples included particulate chlorophyll-a, particulate cyanotoxins (microcystins and saxitoxin), dissolved nutrients (ortho-phosphate, nitrate, nitrite, ammonium), total (whole water) phosphorus and nitrogen, TKN, and particulate nitrogen and were collected as described above. 

#### 4.2.2. Laboratory Analysis of Microcystin and Saxitoxin Samples

Discrete toxin samples were analyzed using enzyme-linked immunosorbent assay (ELISA) for both microcystins and saxitoxins. Microcystins were analyzed using the Envirologix QuantiPlateTM kit (Envirologix, Portland, Me, USA, Cat. No. EP 022, as described in [48]). The Envirologix ELISA kit has been compared with liquid chromatography/mass spectrometry (LC-MS) analysis at University of California, Santa Cruz [48]. The BIOO Scientific MaxSignalTM Saxitoxin (PSP) test kit (BIOO Scientific Corp., Austin, TX, USA, Cat. No. 1034) was used for saxitoxin analysis. These plates provide some cross-reactivity with other toxin congeners, but primarily target STX and dcSTX. Prior to analysis, the sample filters were extracted in 3 mL of Milli-Q™ water, sonicated for 30 s to ensure cell disruption, and centrifuged for 10 min at 2147 g (as described in [88]). The extract was then analyzed per the manufacturer’s instructions for both toxins (Envirologix: http://www.envirologix.com/wp-content/uploads/2016/04/EP022Microcystin.pdf and BIOO: http://www.biooscientific.com/Phycotoxin-test-kits/MaxSignal-Saxitoxin-PSP-ELISA-Test-Kit). The limit of detection for the microcystin and saxitoxin ELISA’s are 0.10 ppb and 0.4 ppb respectively. 

SPATT samples were analyzed at the University of California, Santa Cruz for four microcystin congeners (MCY-LA, MCY-LR, MCY-RR, MCY-YR) by LC-MS with electrospray ionization (ESI) with selected ion monitoring (SIM) on an Agilent 6130 with a Phenomenex Kinetix (100 X 2.10) C18 column. The method was adapted from [89] with minor modifications to account for the choice of column and LCMS/SIM instead of tandem mass spectrometry [48]. The samples were prepared as described in [48]. Analysis included replicates and matrix-additions, with the quantification based on external standards. The method detection limit was 0.05 ng g^−1^ for all congeners. Percent recovery is reported in [48], and was ~58–100% for the congeners using a standardized recovery method, with MCY-RR being lowest followed by MCY-LR (~88%), MCY-YR (~100%), and MCY-LA (~100%).

#### 4.2.3. Laboratory Analysis of Discrete Samples

Chlorophyll-a samples were analyzed at the Southern California Coastal Water Research Project following the method EPA 445.0. Nutrient samples were analyzed at Physis Environmental Laboratories, in Anaheim, California using the following methods: Total phosphorus (SM 4500-P E), dissolved nitrate + nitrite (EPA 300.0), TKN (EPA 351.2), dissolved ortho-phosphate (EPA 300.0) and alkalinity (SM 2320B).

### 4.3. Surveys of Lakes, Reservoirs, and Coastal Lagoons

#### 4.3.1. Site Selection, Sampling Approach and Discrete Sample Collection

There were two types of surveys conducted of lakes, reservoirs and coastal lagoons: (a) a screening assessment survey in 2013; and (b) an ad hoc bloom event response survey in 2014. The screening assessment study was conducted during the summer and fall in 2013 in San Diego and Orange Counties (the two southern-most counties in coastal California). The sites were chosen based on several criteria including the following: (1) high number of beneficial uses of the water body; (2) high frequency of use by the public; (3) impaired nutrient status based on the Clean Water Act Section 303(d) list; and (4) high risk of cyanobacterial blooms. There were 19 sites sampled three times in the screening assessment survey in 2013 between July and October including 10 lakes and reservoirs and 9 estuaries (see Table 4 for list of sites).

The screening assessment survey in 2013 collected discrete samples from composite surface water samples as described in the Depressional Wetlands Assessment (see Section 4.2.1 Site Selection and Sampling Approach for Discrete Samples). SPATT bags were also deployed for two 1-month intervals, typically from July to August and from August to September, as described above in the Depressional Wetlands Assessment (Section 4.2). Discrete samples were collected at the time of each SPATT deployment and retrieval, for a total of three discrete sampling events per site. Discrete samples were collected as described above (Section 4.2.1 Site Selection and Sampling Approach for Discrete Samples), and consisted of particulate chlorophyll-a, particulate microcystins and particulate pigments (phycoerythrin and phycocyanin pigments). The pigment samples were collected on 1 µm Whatman polycarbonate filters, covered in aluminum foil and frozen immediately. In situ measurements of temperature, pH, dissolved oxygen (DO), conductivity, alkalinity and salinity were also collected at each site.

Due to reports of visible blooms, an ad hoc bloom event response survey was conducted in the summer of 2014 in San Diego, Orange and Riverside Counties. Discrete samples of surface water were collected in glass bottles from one location at the edge of the lake within the visible bloom. There were 17 lakes sampled between May and August for discrete samples of cyanobacterial species identification (whole water samples) and particulate cyanotoxins (see Table 5 for list of sites). The samples for cyanobacterial identification were stored in an incubator overnight and analyzed live the following day. The particulate cyanotoxin samples were filtered onto a Whatmann GF/F and frozen immediately. The samples for cyanobacterial identification were used to determine if cyanotoxin sample analysis was needed due to potential toxin-producing genera present in the samples. 

The cyanotoxin discrete samples collected from four lakes sampled in May 2014 (Lake Elsinore, Canyon Lake, San Joaquin Marsh, and Lake Menifee) entailed analysis of whole water, and therefore results are representative of total toxins (both particulate and dissolved). 

#### 4.3.2. Laboratory Analysis of Cyanotoxin Samples

The discrete samples collected from the screening assessment study of lakes, reservoirs and coastal lagoons during 2013 and most of the ad hoc bloom response survey in 2014 were analyzed for 9 microcystin congeners (MCY-LA, MCY-LR, MCY-RR, MCY-YR, MCY-LW, MCY-LY, MC-desmethyl-LR, MC-desmethyl-RR, and MCY-LF) and nodularin at the California Fish and Wildlife Water Pollution Control Lab (WPCL) using LC-ESI-MS/MS as described in [89]. The discrete cyanotoxin samples collected at Lake Menifee, Lake Elsinore, Canyon Lake and San Joaquin Marsh were analyzed at University of California Santa Cruz for four microcystin congeners (MCY-LA, MCY-LR, MCY-RR, MCY-YR), anatoxin-a and cylindrospermopsin, by liquid chromatography/mass spectrometry (LCMS) with electrospray ionization (ESI) with selected ion monitoring (SIM) on an Agilent 6130 with a Phenomenex Kinetix (100 X 2.10) C18 column. The method was adapted from [89] with minor modifications to account for the choice of column and LCMS/SIM instead of tandem mass spectrometry [48].

SPATT samples were analyzed at University of California, Santa Cruz as described previously (see Section 4.2.2 Laboratory Analysis of Microcystin and Saxitoxin Samples).

#### 4.3.3. Discrete Sample Analysis 

Cyanobacterial genera and species identification samples were collected from all of the ad hoc bloom event response samples (2014) and analyzed at the University of Southern California. Samples were examined live within one day of collection. Briefly, homogenized water was aliquoted into shallow plastic tissue culture dishes and allowed to settle for approximately 30 min. The subsamples (5 mL) were viewed with an Accu-Scope (Commack, New York, NY, USA) 3032 inverted microscope and cyanobacteria were identified to genus and when possible, the species was identified. The San Joaquin Marsh sample was viewed using the CellScope Aquatic (Berkeley, CA, USA) field microscope at SCCWRP. 

Phycoerythrin and phycocyanin pigment samples were sent to the laboratory of Dr. Gregory Boyer at the State University of New York and analyzed using a Milton-Roy MR3000 UV-VIS Spectrophotometer.

### 4.4. Statistical Analysis 

The relationships between environmental variables, microcystins and chlorophyll-a were determined for the depressional wetlands assessment and the screening assessment survey in 2013 (no statistical analysis was performed on the ad hoc bloom response survey in 2014). Statistical packages of RStudio, (version 0.96.122 2009-2011, Boston, MA, USA) were used and the significance was set at 0.05 for all statistical analysis. Logistic regression was used to determine if chlorophyll-a (log 10 transformed), was a significant predictor of microcystin and additional environmental variables were included when data were available. The variables from the depressional wetlands assessment include the following: Landscape disturbance indicators (including percentage of agriculture, percentage of urban, percentage of agriculture and urban, impervious cover and road density) derived from the 2001 National Land Cover Database, total nitrogen and phosphorus, temperature, pH, alkalinity, elevation, conductivity and 3 condition indices developed for depressional wetlands [68]; (1) assemblages of macroinvertebrates [90]; (2) benthic diatom index [34]; and (3) the California Rapid Assessment Method (CRAM), which is a visual assessment of the plants and physical habitat [91]. 

The environmental variables used in the screening assessment survey in 2013 included alkalinity, total nitrogen, total phosphorus, cyanobacteria pigments (phycocyanin and phycoerythrin), temperature, salinity, conductivity, DO, and pH.

## Figures and Tables

**Table 1:** List of study sites from the depressional wetlands assessment survey including year sampled, site name, water regime, wetland function and region location. The site numbers correspond to the numbers listed in Figure 1 and Figure 2 and the asterisk (*) indicates sites in San Diego that were revisited and more intensely sampled in summer 2012.

Year	Site Name	Site Number	Water Regime	Wetland Function	Region
2013	Admiral Baker Golf Course	1	Perennial	Golf course	San Diego
2011	Agua Hedionda	2	Perennial	Habitat/stormwater	San Diego
2011	Ballona freshwater marsh	3	Perennial	Habitat/stormwater	Los Angeles
2013	Big Canyon Golf Course	4	Perennial	Golf course	Riverside
2012, 2013	Buena Vista Park	5	Perennial	Habitat/stormwater	San Diego
2011, 2013	Calico Ranch Rd Julian	6	Seasonal	Habitat/stormwater	San Diego
2012	Calle Roxanne Fallbrook	7 *	Perennial	Private property	San Diego
2011	Circle X	8	Seasonal	Stock pond	Los Angeles
2013	Costa del Sol Golf Course	9	Perennial	Golf course	San Diego
2012	Covina flood control basin	10	Perennial	Flood control	Los Angeles
2013	Creek Hollow Ranch	11	Seasonal	Habitat/stormwater	San Diego
2011	Dairy mart	12	Perennial	Habitat/stormwater	San Diego
2011	De La Garrigue Rd	13	Perennial	Stock pond	Los Angeles
2012	Dominguez gap west basin	14	Perennial	Flood control	Los Angeles
2012	Emerald Isle Golf Course	15 *	Perennial	Golf course	San Diego
2012	Euclid Edison	16	Seasonal	Private property	Riverside
2011	Foss Lake Alkali Marsh	17	Seasonal	Habitat/stormwater	San Diego
2011	Guajome Lake	18	Perennial	Habitat/stormwater	San Diego
2012	Harbor Lake (Lake Machado)	19	Perennial	Habitat/stormwater	Los Angeles
2011	Harbor Lakes 2	20	Perennial	Habitat/stormwater	Los Angeles
2012	Hemet Golf Course	21	Perennial	Golf course	Riverside
2011	Irvine Turtle Ridge	22	Perennial	Flood control	Riverside
2013	IRWD San Joaquin Pond 5	23	Perennial	Habitat/stormwater	Riverside
2011	Laguna Lake	24	Perennial	Habitat/stormwater	Riverside
2012	Links at Summerly	25	Seasonal	Golf course	Riverside
2012	Lyons Valley Rd Jamul	26 *	Seasonal	Private property	San Diego
2011	Madrona Marsh	27	Seasonal	Habitat/stormwater	Los Angeles
2012	Manchester Ave Encinitas	28	Seasonal	Private property	San Diego
2012	Michelson Marsh	29	Perennial	Habitat/stormwater	Riverside
2012	Mountain View Golf Course	30	Perennial	Golf course	Los Angeles
2011	Murrieta	31	Seasonal	Habitat/stormwater	Riverside
2011	Nicholas Flat pond	32	Perennial	Habitat/stormwater	Los Angeles
2011, 2012	Olive Hill Road Fallbrook	33 *	Perennial	Private property	San Diego
2013	O’Neill Park pumping station	34	Perennial	Flood control	San Diego
2012	Pala Rey Ranch	35 *	Perennial	Private property	San Diego
2012	Palm Lake Golf Course	36	Perennial	Golf course	Los Angeles
2011, 2012	Palomar Airport Rd	37	Seasonal	Habitat/stormwater	San Diego
2012	Pico Rivera Municipal Golf Course	38	Perennial	Golf course	Los Angeles
2012	Prado Recreation Inc	39	Perennial	Habitat/stormwater	Riverside
2013	Robinson Ranch Golf Course	40	Perennial	Golf course	Los Angeles
2013	San Diego River Ponds P11BA Santee Recreation Lakes	41	Perennial	Recreation	San Diego
2011	San Diego River Santee	42	Perennial	Habitat/stormwater	San Diego
2012	San Dieguito River Calle Ambiente	43 *	Perennial	Habitat/stormwater	San Diego
2012	Santee Lakes Recreation Preserve Lake #7	44 *	Perennial	Recreation	San Diego
2013	Santo Rd San Diego	45	Perennial	Habitat/stormwater	San Diego
2013	Simi Hills Golf Couse	46	Perennial	Golf course	Los Angeles
2013	Sims Pond (Los Cerritos)	47	Perennial	Habitat/stormwater	Los Angeles
2012	Sunsol nursery	48	Perennial	Habitat/stormwater	San Diego
2012	Sweetwater Authority El Tae Rd	49 *	Seasonal	Private property	San Diego
2012	Tumble Creek Lane	50	Perennial	Private property	San Diego
2013	Vista Valencia Golf Course	51	Perennial	Golf course	Los Angeles
2011	Zuniga Marsh	52	Seasonal	Stock pond	Los Angeles

**Table 2:** Summary of depressional wetlands discrete toxin sample results collected during the spring assessments in 2011, 2012 and 2013 throughout Southern California.

Year Sampled	Percent of Sites Microcystins Detected	Percent of Sites Saxitoxins Detected	Range of Concentrations of Microcystins (µg L^−1^)
2011	12.5	NA	bd–2.5
2012	47	5	bd–0.45
2013	12.5	0	bd–22
All years combined	25.4	2	

bd = below the method detection limit, NA = not analyzed.

**Table 3:** The percentage of depressional wetlands sites where microcystins were detected based on discrete samples and SPATT samples, for collection sites in San Diego County, sampled in 2012.

Season	Percent of Sites with Microcystins Detected in Discrete Samples	Percent of Sites with Microcystins Detected in SPATT Samples
Spring	60	Not collected
Summer	29	83
Fall	29	

**Table 4:** List of study sites and concentrations of microcystins detected from both discrete and SPATT samples collected in the screening assessment survey of lakes, reservoirs and coastal lagoons in 2013. Sites for which microcystins were detected throughout the entire study period are in bold and italics. The site numbers provided correspond to Figure 3.

Site Name	Site Number on Map	Range of Total Microcystins Detected from Discrete Samples (µg L^−1^)	Range of Total Microcystins Detected from SPATT Samples (ng g^−1^)
Lakes and Reservoirs			
***Lake Henshaw***	3	bd–0.1	1.3–2.1
Cuyamaca Reservoir	1	bd	0.9
Lower Otay Reservoir	8	bd	NA
Lake Murray	6	bd	bd–8.5
Morena Reservoir	9	0.02–23.6	44.7
Vail Lake	10	bd–2.1	bd–13.3
***Lake Hodges***	4	bd	0.5–2.7
Lake Sutherland	7	bd	bd–44.3
El Capitan Lake	2	bd	bd–1.6
Lake Miramar	5	bd–0.1	5.6–7.0
Estuaries			
***San Elijo Lagoon***	17	bd	1.2–1.5
***San Elijo Pond***	18	bd	2.3–4.5
Los Penasquitos Lagoon	11	bd	bd–2.3
San Diego Bay near Silver Strand Bikeway	14	bd	bd–12.2
San Diego Bay near Sweetwater	15	bd	bd–0.2
***San Diego Bay near Naval Training Center***	13	bd	3.2–6.0
San Diego River Estuary	16	bd	bd–2.4
Mission Bay	12	bd	bd–15.1
***Tijuana River Estuary***	19	bd–0.09	2.7–100.8

bd = below the method detection limit; NA = not analyzed.

**Table 5:** List of study sites from the ad hoc bloom response survey in 2014 and discrete sample results for cyanobacterial identification and total microcystins (MCY) reported in µg L^−1^. Sites in bold exceeded the California health advisory thresholds for recreational exposures.

Name	Site Number on Map	Cyanobacterial Genera and Species Identification	MCY
Barrett Lake	20	*Cylindrospermopsis raciborskii*, *Cylindrospermopsis* spp., *Anabaena* spp.	bd
Canyon Lake	25	*Anabaena* sp., *Raphidopsis* sp.	0.01
Chollas Reservoir		Low abundance of non-nitrogen fixing filaments	NA
Discovery Lake		*Planktothrix* sp., *Anabaena variabilis*, *Anabaena spiroides*, *Cylindrospermopsis* sp.	NA
Guajome Lake		*Cylindrospermopsis* sp., *Planktothrix* sp.	NA
Harveston Lake *^,¥^	21	Sparce *Microcystis* sp.	10.0
Lake Barbara	22	no cyanobacteria observed	bd
Lake Elsinore ^§^	26	*Anabaena* sp., *Cylindrospermopsis* spp.	0.01
Lake Hodges	4	*Anabaena* sp.	bd
Lake Henshaw, outflow	3	*Microcystis* sp.	bd
Lake Menifee ^§^	27	*Cylindrospermopsis* sp. *Raphidopsis* sp.	bd
Lake Morena		Mainly eukaryotes, shoreline dominated by *Microcystis* spp.	NA
Lake Poway		Sparse *Microcystis* sp. colonies	NA
Lake Sutherland	7	*Microcystis* sp.	bd
Lindo Lake *	23	*Planktothrix* spp., *Anabaena variabilis, Anabaena* spp. *Cylindrospermopsis* spp., *Microcystis* sp.	2.5
**San Joaquin Marsh**	28	*Microcystis* sp.	36,549
**Santee Lake #5**	24	*Microcystis* sp., *Cylindrospermopsis* sp. *Cylindrospermopsis raciborskii* and *Anabaena spiroides*	11.7

NA = not analyzed; bd = below the method detection limit. * = Sampled in both June and August. ^¥^ = toxin results for June only; cyanobacteria identification genera results for August only. ^§^ = sites exceeded CA health advisory thresholds for either cylindrospermopsin or anatoxin-a concentrations.

## Supplementary Materials

The following are available online at www.mdpi.com/2072-6651/9/7/231/s1, Table S1: SPATT sample results for all microcystin congeners (in ng g^−1^) analyzed from the depressional wetlands sites that were revisited in summer and fall 2012 in San Diego, Table S2: SPATT results for all microcystin congeners (in ng g^−1^) analyzed from the 2013 screening assessment survey of lakes, reservoirs and coastal lagoons, Table S3: Discrete sample results for all microcystin congeners analyzed (in µg L^−1^) from the screening assessment survey of lakes, reservoirs and coastal lagoons in 2013, Table S4: Discrete sample results of all microcystin congeners (in μg L^−1^) analyzed from the ad hoc bloom event response survey in 2014.
