# Supplementary material for: Microcystin Prevalence throughout Lentic Waterbodies in Coastal Southern California
Source: Toxins (Basel). 2017 Jul 22;9(7):231. doi: 10.3390/toxins9070231 (PMC5535178; doi:10.3390/toxins9070231)
Supplement: Supplementary file 1 [file toxins-09-00231-s001.pdf]

Supplementary Materials: Microcystin Prevalence throughout Lentic Waterbodies in Coastal Southern California

Meredith D. A. Howard [1,*], Carey Nagoda [2], Raphael M. Kudela [3], Kendra Hayashi [3], Avery Tatters [4], David A. Caron [4], Lilian Busse [5], Jeff Brown [1], Martha Sutula [1], Eric D. Stein [1]

Table S1. SPATT sample results for all microcystin congeners (in ng g^{-1}) analyzed from the depressional wetlands sites that were revisited in summer and fall 2012 in San Diego.

Site Name	Site Number on Map	Total MCY	MCY-LR	MCY-RR	MCY-YR	MCY-LA
Calle Roxanne Fallbrook	7	0.05	bd	0.05	bd	bd
Emerald Isle Golf Course	15	0.37	bd	0.37	bd	bd
Lyons Valley Rd Jamul	26	7.36	7.36	bd	bd	bd
Olive Hill Road Fallbrook	33	0.17	bd	0.17	bd	bd
Pala Rey Ranch	35	2.18	2.18	bd	bd	bd
San Dieguito River Calle Ambiente	43	0.06	bd	0.06	bd	bd
Santee Lakes Recreation Preserve Lake #7	44	97.02	81.18	bd	bd	15.84

bd = below the method detection limit.

Table S2. SPATT results for all microcystin congeners (in ng g^{-1}) analyzed from the 2013 screening assessment survey of lakes, reservoirs and coastal lagoons. Concentrations on the top correspond to the summer samples from August and the bottom concentrations represent the early fall samples in late September/early October.

Site Name	Site Number on Map	Total MCY	MCY-LR	MCY-RR	MCY-YR	MCY-LA
Lakes and Reservoirs						
Lake Henshaw	3	2.1<br>1.3	bd<br>bd	2.1<br>1.3	bd<br>bd	bd<br>bd
Cuyamaca Reservoir	1	0.9<br>NA	bd<br>NA	0.9<br>NA	bd<br>NA	bd<br>NA
Lower Otay Reservoir	8	NA<br>NA	NA<br>NA	NA<br>NA	NA<br>NA	NA<br>NA
Lake Murray	6	8.5<br>bd	bd<br>bd	8.5<br>bd	bd<br>bd	bd<br>bd
Morena Reservoir	9	NA<br>44.7	bd<br>30.8	bd<br>13.9	bd<br>bd	bd<br>bd
Vail Lake	10	bd<br>13.3	bd<br>12.6	bd<br>0.7	bd<br>bd	bd<br>bd
Lake Hodges	4	2.7<br>0.5	bd<br>bd	2.7<br>0.5	bd<br>bd	bd<br>bd
Lake Sutherland	7	44.3<br>bd	bd<br>bd	16.3<br>bd	bd<br>bd	28<br>bd
El Capitan Lake	2	1.6<br>bd	bd<br>bd	1.6<br>bd	bd<br>bd	bd<br>bd
Lake Miramar	5	7.0<br>5.6	bd<br>4.9	6.0<br>0.6	bd<br>bd	1.0<br>bd
Estuaries						
San Elijo Lagoon	17	1.5<br>1.2	bd<br>bd	bd<br>1.2	bd<br>bd	1.5<br>bd
San Elijo Pond	18	4.5<br>2.3	bd<br>bd	4.5<br>2.3	bd<br>bd	bd<br>bd
Los Penasquitos Lagoon	11	bd<br>2.3	bd<br>bd	bd<br>2.3	bd<br>bd	bd<br>bd
San Diego Bay near Silver Strand Bikeway	14	12.2<br>bd	bd<br>bd	7.7<br>bd	bd<br>bd	4.5<br>bd
San Diego Bay near Sweetwater	15	bd<br>0.2	bd<br>bd	bd<br>0.2	bd<br>bd	bd<br>bd
San Diego Bay near Naval Training Center	13	3.2<br>6.0	bd<br>1.6	3.2<br>4.4	bd<br>bd	bd<br>bd
San Diego River Estuary	16	bd<br>2.4	bd<br>bd	bd<br>2.4	bd<br>bd	bd<br>bd
Mission Bay	12	bd<br>15.1	bd<br>12.9	bd<br>2.2	bd<br>bd	bd<br>bd
Tijuana River Estuary	19	100.8<br>2.7	81.6<br>bd	bd<br>2.7	bd<br>bd	19.2<br>bd

bd = below the method detection limit, NA = not analyzed.

Table S3. Discrete sample results for all microcystin congeners analyzed (in μg L^{-1}) from the screening assessment survey of lakes, reservoirs and coastal lagoons in 2013.

Site Name		Total MCY	LA	LR	RR	YR	LW	LY	des-LR	des-RR	LF *
Vail Lake	June *	2.1	bd	1.3	0.2	0.5	NA	NA	NA	NA	NA
	July	bd									
	Sept	bd									
Lake Henshaw	June *	0.1	bd	0.1	0.02	bd	NA	NA		NA	
	July	bd									
	Aug	0.08	bd	0.08	bd	bd	bd	bd	bd	bd	bd
	Sept	bd									
Morena Reservoir	July	0.02	bd	0.02	bd	bd	bd	bd	bd	bd	bd
	Aug	6.1	0.1	3.1	0.3	1.9	0.1	<0.1	bd	0.04	0.3
	Sept	23.6	10.1	9.9	1.2	1.3	0.2	bd	0.6	0.1	bd
Tijuana River Estuary	July	0.09	bd	bd	bd	0.09	bd	bd	bd	bd	bd
	Aug	0.05	bd	bd	bd	0.05	bd	bd	bd	bd	bd
	Sept	bd									

Abbreviations above are as follows: Microcystin-LA = LA, Microcystin-LR = LR, Microcystin-RR = RR, Microcystin-YR = YR, Microcystin-LW = LW, Microcystin-LY = LY, Microcystin-desmethyl-LR = des-LR, Microcystin-desmethyl-RR = des-RR, Microcystin-LF = LF. bd = below the method detection limit (see methods). * These samples were collected during the site reconnaissance and analyzed at UCSC as described above for the Riverside samples. Therefore, only 4 MCY congeners were analyzed. NA = not analyzed.

Table S4. Discrete sample results of all microcystin congeners (in μg L^{-1}) analyzed from the ad hoc bloom event response survey in 2014.

Name	Total MCY	MCY-LR	MCY-RR	MCY-YR	MCY-LA	MCY-desmethyl-RR	MCY-desmethyl-LR	MCY-LF	MCY-LW	MCY-LY
Canyon Lake	0.016	0.014	0.002	bd	bd	NA	NA	NA	NA	NA
Harveston Lake	10.0	2.23	4.49	2.99	bd	0.7	0.31	bd	bd	bd
Lake Elsinore	0.01	bd	0.004	bd	0.007	NA	NA	NA	NA	NA
Lindo Lake (June)	2.56	1.26	0.844	bd	bd	bd	bd	bd	bd	0.46
Lindo Lake (August)	2.44	1.11	0.55	0.06	bd	0.08	0.08	bd	bd	0.56
Menifee Lake	bd	bd	bd	bd	bd	NA	NA	NA	NA	NA
Santee Lake	11.71	7.69	bd	bd	3.3	bd	0.5	0.06	0.09	0.09
San Joaquin Marsh	36,549	32,540	2,487	721	801	NA	NA	NA	NA	NA

Abbreviations above are as follows: Microcystin-LA = LA, Microcystin-LR = LR, Microcystin-RR = RR, Microcystin-YR = YR, Microcystin-LW = LW, Microcystin-LY = LY, Microcystin-desmethyl-LR = des-LR, Microcystin-desmethyl-RR = des-RR, Microcystin-LF. bd = below the method detection limit and NA = not analyzed.
